# Ovarian fibrosarcoma: A single-institution experience and a review of the literature

**DOI:** 10.1186/s13048-020-00749-x

**Published:** 2020-12-08

**Authors:** Ting-Ting Sun, Ning-Hai Cheng, Dong-Yan Cao, Peng Peng

**Affiliations:** Department of Obstetrics and Gynecology, Peking Union Medical College, Peking Union Medical College Hospital, Chinese Academy of Medical Science, Shuaifuyuan No. 1, Dongcheng District, 100730 Beijing, China

**Keywords:** Ovarian fibrosarcoma, Pathology, Treatments, Prognosis

## Abstract

Ovarian fibrosarcoma is an extremely rare and malignant sex cord-stromal tumor. Due to its low incidence and poor prognosis, until now, very few cases have been reported, and most of the reported cases have been sporadic. Therefore, the treatments and prognostic factors of ovarian fibrosarcoma are still debatable. Here, we report 5 cases of ovarian fibrosarcoma that presented at Peking Union Medical College Hospital over the past 20 years. The 5 patients were 41, 51, 54, 76, and 76 years of age when initial symptoms of pelvic mass or pain appeared. On ultrasound, this disease usually manifests as unilateral pelvic masses, within which uneven echo enhancement and some blood flow signals are observed. No significant increase was observed in the levels of preoperative tumor markers, such as serum CA125 and sex hormones. The final diagnosis depends on postoperative histopathological results since these tumors are easily misdiagnosed when intraoperative frozen sections are used for examination. Pathologic examinations showed that the tumor cells were spindle-shaped with moderate to severe atypia and high mitotic counts. The immunohistochemistry profile is not specific, but the positive rate of Ki-67 was consistent with the degree of malignancy and the prognosis of patients with this tumor. In addition, the tumor may also be positive for Vimentin, α-inhibin, SMA, estrogen receptor and progesterone receptor. Significant differences were observed in the surgical methods used, and no unified chemotherapy regimen has been established. The overall survival was > 15, >7, > 6, <1, and < 1 year for each patient. After reviewing the literature, evidence-based large-scale case studies were lacking. For treatments, complete cytoreductive surgery plus regimens typically used against malignant sex cord-stromal tumors, as described in the NCCN guidelines, are recommended. Due to its low incidence, both multicenter clinical studies and molecular studies are required to provide gynecologists with a better understanding and guidance for future management of patients with ovarian fibrosarcoma.

## Introduction

Ovarian fibrosarcoma is a considerably rare neoplasm, as this tumor type accounts for less than 1% of all ovarian malignancies [1]. According to previous reports, ovarian fibrosarcoma may originate from stromal cells that surround the sex cord of ovarian follicles and the fibrous components of the ovarian phylum, or from the malignant transformation of a benign ovarian fibroma [2]. Ovarian fibrosarcoma can occur at any age, although most of these tumors appear in menopausal and postmenopausal women and are associated with an extremely poor prognosis. Some previous studies have reported on ovarian fibrosarcoma, most of which had a 2-year overall survival rate of approximately 55.9% [3]. The diagnosis is primarily based on pathological examination, and immunohistochemistry helps to confirm the diagnosis. Due to its extremely low incidence, very few cases have been reported, and most of the reported cases are sporadic. Here, we present 5 cases of ovarian fibrosarcoma that presented at Peking Union Medical College Hospital (PUMCH) during the past 20 years. The accumulation of these cases is useful for future diagnosis and treatments.

## Case reports

### Case 1

A 57-year-old woman was admitted to our clinic with an abdominal mass that had appeared 2 weeks earlier, but she did not experience symptoms of abdominal pain and distension. She had been postmenopausal for 3 years and had no vaginal bleeding after menopause. Vaginal and abdominal examinations confirmed the presence of a cystic mass, with a diameter of 6–7 cm and no pressing pain. Vaginal ultrasonography revealed a mass 9.6*8.2*5.2 cm in diameter that was thickly encapsulated with a liquid and irregular mass in the left ovarian region. The interior of the cyst contained several septa, with medium to strong echo bulges on the septa, the largest of which was 1.8*1.1 cm in size. A slight blood flow signal could be seen on the septum, and a liquid area with a maximum depth of 4.6 cm could be seen in the pelvic cavity. Before surgery, the levels of serum tumor markers were within normal limits.

During the laparotomy, an irregular, multilocular, cystic mass 8*10 cm in diameter was found in the right ovarian region, and no metastatic lesions were observed. Total abdominal hysterectomy (TAH) plus bilateral adnexectomy was performed. The pathologic diagnosis of the mass was a well-differentiated sarcoma of the right ovary (fibrostromal sarcoma or fibrosarcoma). The immunohistochemical results were as follows: Vimentin (+), SMA (+), PR (±), ER (-), CD10 (-), Caldesmon (-), Melan (-), α-inhibin (-).

The tumor was categorized as an International Federation of Gynecology and Obstetrics (FIGO) stage I C. The patient was given 2 cycles of systemic chemotherapy consisting of Cisplatin + Vincristine + Bleomycin (PVB) at 3-week intervals. After chemotherapy, the patient was followed-up regularly, and no recurrence was seen 15 years after surgery.

### Case 2

A 41-year-old female was admitted with an abdominal mass that had appeared 2 months earlier. This patient reported no additional complaints, such as abdominal pain or distension. She had normal menstruation and no abnormal vaginal bleeding. Upon abdominopelvic examination, a firm, unmovable mass 5*6 cm in diameter without tenderness was detected on the posterior left side of the uterus. Vaginal ultrasonography revealed a heterogeneous, left ovarian mass with a clear boundary and a diameter of 6.1*5.5*4.6 cm. No signs of metastasis, lymph node enlargement or ascites were observed. No obvious blood flow signal was detected. The levels of serum tumor markers were within normal limits with the exception of a slightly elevated carcinoma antigen (CA) 125 level (40.4 U/mL). By magnetic resonance imaging (MRI), a heterogeneous mass 48*62*60 mm in diameter that was closely related to the uterus was detected on the posterior left side of the uterus. The possibility of subserosal fibroids was considered, whereas an adnexal tumor was not considered.

During laparoscopic exploration, we found a solid mass 7*6 cm in diameter with a clear boundary; the mass originated in the left ovary, and no peritoneal dissemination was observed. The right ovary appeared full and the uterus appeared normal. Ovarian cystectomy, sampling of the right ovary and intraperitoneal washing were performed during surgery. After the mass was cut open, solid granular materials were present. The intraoperative pathological diagnosis was left ovarian follicular fibroma. However, a week later, the final pathologic diagnosis was fibrosarcoma of the left ovary, and the mitotic counts were evaluated > 10 times in 10 high-power fields (HPFs). The immunohistochemical analysis showed AE1/AE3 (-), Calretinin (-), Ki-67 (index 5%), p53 (-), α-inhibin (-). After surgery, the serum CA125 level was 27.1 U/ml.

This case was diagnosed as FIGO stage I A. The patient refused chemotherapy and remained disease-free with normal ovarian function during the 7-year follow-up.

### Case 3

A 76-year-old woman was admitted with abdominal pain and fever. She had been postmenopausal for 28 years without any vaginal bleeding after the onset of menopause. Fourteen years ago, the patient underwent an exploratory laparotomy due to acute abdomen at another hospital. During the operation, 2800 ml of intraabdominal blood clots and non-clotted blood was removed, and a cauliflower-like tumor 3*3*2 cm in diameter with active bleeding was seen around the left tubal umbrella. Complete hysterectomy and bilateral salpingo-oophorectomy and omentectomy were performed. The postoperative pathology report described a diffuse granular follicular cell tumor of the left ovary with invasion of the left fallopian tube. The clinical FIGO stage was II C. After surgery, she received 3 cycles of systemic chemotherapy consisting of Cisplatin + Cyclophosphamide (PC), after which the patient did not return for follow-up.

Four years later (10 years ago), the patient underwent a second exploratory laparotomy at another hospital due to abdominal pain and the presence of a pelvic mass. During the surgery, a solid-cystic mass 3 cm in diameter was observed between the sigmoid colon and the bottom of the left side of the bladder. In addition, a nodule 1 cm in diameter at the bottom of the left side of the bladder bottom and a nodule 0.5 cm in diameter on the surface of the sigmoid colon were seen. The tumor lesions were completely removed, and the postoperative pathological diagnosis was granulosa cell tumor. After surgery, she received 1 cycle of chemotherapy consisting of Taxol + Carboplatin. However, the patient was then lost to follow-up.

The next time, she was admitted for abdominal pain and fever that persisted for 1 day. Ultrasound revealed an irregular, cystic mass 10*8.6 cm in diameter with an unclear boundary in the pelvic cavity; the wall was uneven in its thickness, and the size of the cystic portion was approximately 7.21*6.34 cm. MRI revealed a double capsular structure 88*9*7 cm in diameter on the left side of the pelvic cavity. The mass had a thick wall with a relatively clear edge and was located close to the right bowel. The cephalic size of the lesion was approximately 4*2*3 cm, and the signal of the vesicle was slightly low. Vaginal and abdominal examinations confirmed the presence of a hypertonic, cystic mass with a diameter of 16 cm. The preoperative serum levels of CA125, Estradiol (E2), and Follicle Stimulating Hormone (FSH) were 15.5 U/ml, 25.8 pg/mL and 52.8 mIU/mL, respectively.

We performed exploratory laparotomy and secondary cytoreductive surgery. During the operation, a solid-cystic multilocular mass 10 cm in diameter was observed between the sigmoid colon and the left lateral wall of the bladder. After 600 ml of yellow intracapsular fluid was removed, the mass was completely excised. Then, we performed adhesion decompression, repair of the sigmoid colon, and partial ileotomy anastomosis. The tumor was then completely removed, and the pathologic diagnosis was ovarian fibrosarcoma. Immunohistochemical analysis showed the following: Melan-A (+), Vimentin (+), AE1/AE3 (+/-), CD99 (-), Calretinin (-), α-inhibin (-), Ki-67 index 10%.

After surgery, the patient experienced incomplete intestinal obstruction and received conservative treatment. The patient’s condition gradually improved and she fully recovered after 2 weeks. Since the patient was elderly, she did not receive adjuvant chemotherapy. No signs of recurrence or an increase in the serum E2 level have been observed more than 6 years after surgery.

### Case 4

A 76-year-old woman was admitted with abdominal pain and an abdominal mass. She had been postmenopausal for 28 years and had no abnormal vaginal bleeding. Ultrasound revealed a heterogeneous, irregular, hypoechoic mass without a clear boundary that was located on the upper right-hand side of the uterus, with a diameter of 11.4*13.5*8.6 cm. Punctiform blood flow signals could be seen by Color Doppler Flow Imaging (CDFI). MRI revealed a large solid-cystic mass in the pelvic cavity, and we considered the possibility of malignant lesions in the accessory ovary, except mesenchymal tumors. Vaginal and abdominal examinations confirmed the presence of a hypertonic, cystic mass with a diameter of 12–15 cm and no pressing pain. Before surgery, the serum CA125 level was 593.3 U/ml.

Her medical history included sleep apnea syndrome for more than 10 years that necessitated supplementary positive pressure ventilation at night.

After a multidepartment consultation, surgical contraindications were eliminated, and we performed an exploratory laparotomy. During the operation, approximately 100–200 ml of bloody ascites was observed. A cystic-solid mass with a diameter of approximately 20 cm was seen below the incision. Cauliflower-like tumors with a rich blood supply could also be seen on the surface of the mass. The mass was widely adherent to the surrounding small intestine and colon, the surface of which contained cauliflower-like tumor lesions. The source of the mass and the metastatic tumors was found to be the left accessory ovary, and thus, the left adnexa was removed. The pathology report revealed a malignant spindle cell fibrosarcoma. The mitotic counts were evaluated > 10 times/10 HPFs. Immunohistochemical analysis showed the following: CA125 (-), CD10 (partial+), Desmin (-), Ki-67 (index40%), SMA (+), S-100 (-), Vimentin (+), p53 (-), α-inhibin (-).

Due to her medical complications and older age, the patient refused adjuvant chemotherapy. One week later, she was discharged and returned home. The tumor relapsed 2 months later, and she died within 1 year of the first operation.

### Case 5

A 54-year-old woman was admitted with fever and dull abdominal pain. She had been postmenopausal for 18 months and had no abnormal vaginal bleeding. A physical examination confirmed the presence of a cystic mass 10 cm in diameter on the right posterior of the uterus with clear boundaries, poor activity and no tenderness. The serum levels of CA125 and CA199 were 88.8 U/ml and 40.4 U/ml, respectively. Vaginal ultrasonography revealed a cystic mass full of fine spots that was located in the right ovarian area; the mass had clear boundaries, irregular low echo protrusions on the wall and no blood flow signals on CDFI. A solid mass with clear boundaries and a diameter of 7.3*5.9*6.5 cm was located below the former site of the cystic mass, and abundant arteriovenous blood flow signals could be seen inside and around the mass.

Her medical history included 30 years of dysmenorrhea and 18 years of endometriosis and adenomyosis.

We performed an exploratory laparotomy, and during the operation, a small amount of bloody ascites was observed. We observed that the size of the uterus was approximately equal to that at 6 weeks of pregnancy, and no obvious abnormality was seen in the left ovarian area. A cystic-solid mass with an approximate diameter of 10*15 cm was seen in the right ovarian area. After 600 ml of thin, chocolate-like intracapsular fluid was removed, we observed that the mass was densely adherent to the surrounding pelvic peritoneum and that the rectal pouch was completely closed. The source of the mass was found to be the left accessory ovary, and thus, the left adnexa was completely removed. After the mass was cut open, the contents appeared pale white, crisp and vortex-free and contained a chocolate-like liquid. The intraoperative rapid pathology report described a right ovarian spindle cell tumor with a large necrotic area, which was considered a sex cord-stromal tumor, but a malignant tumor was not excluded. After communicating with the patient’s family, they wanted to wait for the final pathology results before proceeding with additional surgery. Total hysterectomy and bilateral adnexectomy were then performed.

However, 10 days later, the final pathologic diagnosis was fibrosarcoma of the right ovary, with extensive necrosis. The mitotic counts were evaluated > 40 times/10 HPFs. Immunohistochemical analysis showed the following: CD31 (+), Ki-67 (+ 70%), SMA (+), AE1/AE3 (-), CD34 (-), CD117 (-), ER (-), PR (-), Desmin (-). No tumor cells were found during peritoneal washing.

After surgery, the serum levels of CA125 and CA19-9 were 109.0 U/ml and 5.6 U/ml, respectively. The patient was given 1 cycle of systemic chemotherapy consisting of Cisplatin + Epirubicin + Ifosfamide (PEI). After 1 course of chemotherapy, the serum levels of CA125 and CA19-9 were 24.9 U/ml and 4.3 U/ml, respectively.

We then performed a second laparoscopic exploration, adhesion decompression and partial removal of the pelvic mass 5 weeks after the first surgery and 3 weeks after chemotherapy. During the surgery, a hard, solid mass approximately 5 cm in diameter was palpable at the top of the stump and was located below the adhesion of the bladder and rectum. The surface of the mass was not visible. No obvious tumor lesions were seen on the pelvic peritoneal surfaces and visceral surfaces. Due to the tight adhesion, separation was extremely difficult, and the mass did not have a capsule or boundaries. Only 2/3 of the tumor was removed, and after the mass was opened, solid, brittle gray material was present.

The pathology report still described a fibrosarcoma. One week after the second surgery, the serum level of CA125 was 35.1 U/ml, and she received 1 cycle of chemotherapy consisting of Taxol + Carboplatin (TC). After 1 course of TC, the serum level of CA125 was 32.9 U/ml, after which the chemotherapy ended, and the patient died within 1 year after surgery (Table 1).

**Table 1:** Clinical characteristics of 5 patients with ovarian fibrosarcoma

NO.	Case 1	Case 2	Case 3	Case 4	Case 5
Age, (year)	57	41	76	76	51
Menopausal status	Yes	No	Yes	Yes	Yes
Gravidity/ Parity	1/1	2/1	4/4	1/1	1/1
Clinical presentations	Mass	Mass	Mass, pain, fever	Mass, pain	Mass, pain, fever
Ultrasound	Thickly capsulated, 9.6*8.2*5.2 cm, irregular, left; septa inside; small amount of blood flow signal; liquid depth 4.6 cm.	Heterogeneous, left, clear boundary, 6.1*5.5*4.6 cm.	Irregular, cystic, 10*8.6 cm, unclear boundary, uneven in thickness.	Heterogeneous, irregular, hypoechoic, unclear boundary, 11.4*13.5*8.6 cm; punctiform blood flow signals; liquid depth 1.6 cm.	Solid, clear boundaries, 7.3*5.9*6.5 cm, abundant arteriovenous blood flow signals.
Size of mass, cm	8*10	7*6	10	20	10*15
Side of mass	Right	Left	Left	Left	Left
CA 125, U/ml (normal range ≤ 35 U/ml)	9.9	40.4	15.5	593.3	88.8
Surgery	TAH + BSO	Ovarian cystectomy + sampling of right ovary.	Secondary cytoreductive surgery	Left adnexectomy	TAH + BSO; Partial removal of pelvic mass
FIGO Stage	IC	IA	-	IIIC	II
Intraoperative pathology	NA	Left ovarian follicular fibroma	NA	NA	Sexostromal tumor, not excluding malignant tumor.
Final pathology	Well-differentiated fibrosarcoma	Fibrosarcoma	Fibrosarcoma	Fibrosarcoma	Fibrosarcoma
Mitotic counts/ HPF	-	> 10	-	> 10	> 40
Ki-67	-	5%	10%	40%	70%
Immunohistochemistry	Vimentin (+), SMA (+), PR (±), ER (-), CD10 (-), Caldeson (-), Melan (-), α-inhibin (-).	AE1/AE3 (-), Calretinin (-), Ki-67 (index 5%), p53 (-), α-inhibin (-).	Melan-A (+), Vimentin (+), AE1/AE3 (+/-), CD99 (-), Calretinin (-), α-inhibin (-), Ki-67 (index10%).	CA125 (-), CD10 (partial+), Desmin (-), Ki-67 (index40%), SMA (+), S-100 (-), Vimentin (+), p53 (-), α-inhibin(-).	CD31(+), Ki-67(+ 70%), SMA(+), AE1/AE3(-), CD34(-), CD117(-), ER(-), PR(-), Desmin(-).
Adjuvant therapy	PVB*2	No	No	No	PEI; TC
Relapse	No	No	No	Yes	Yes
OS, y	> 15	> 7	> 6	< 1	< 1

*Abbreviations*: *y* year, *cm* centimeter, *CA-125* cancer antigen 125, *FIGO* Federation International Gynecology Obstetrics, *PVB*, Cisplatin + Vincristine + Bleomycin, *PEI *Cisplatin + Epirubicin + Ifosfamide, *TC *Taxol + Carboplatin, *OS *overall survival

## Discussion

### Clinical features

Ovarian sex cord-stromal tumors account for 4.3% of all ovarian tumors, and of these, fibroid tumors are the most common type and are usually benign [2]. Ovarian fibroid tumors consist of fibroma, rich cell fibroma and fibrosarcoma. Ovarian fibrosarcoma is very rare, and most previously published studies were case reports. This tumor may be a primary tumor or may result from the malignant transformation of a fibroma. In case 3, the patient received two surgeries before the third surgery because of a pelvic mass. The first two surgeries both resulted in a diagnosis of ovarian granulosa cell tumor, while the third surgery resulted in a diagnosis of fibrosarcoma. We are unsure about the accuracy of previous pathology reports and wonder whether in those reports the fibrosarcoma was a primary tumor or the result of a malignant transformation of a fibroma.

In the 5 cases described above, the patients were 41, 51, 54, 76, and 76 years of age when the initial symptoms of pelvic mass or pain appeared, which is in accordance with previous reports. According to the literature, ovarian fibrosarcoma is common in older women, with a median age of 49 years [3]. Clinically, the manifestations of this tumor are nonspecific, and most patients have a pelvic mass or abdominal pain as the first notable symptom. The ovarian masses often appear as large solid tumors that range in size from 5 to 23 cm with an average size of 11.5 cm [4]. Patients who present with symptoms are usually at an advanced disease stage. Similarly, in our cases, the manifestations on ultrasound are usually unilateral pelvic masses, within which are uneven echo enhancement and larger solid areas. Blood flow signals may be abundant, but no other specific ultrasound characteristics are observed. Ovarian fibrosarcomas also lack specific tumor markers. After a review of the literature and the cases described above, no significant increase in the levels of preoperative tumor markers, such as serum CA125 and sex hormones, was observed. Therefore, the preoperative diagnosis and recurrence monitoring of ovarian fibrosarcoma primarily depend on imaging examinations.

### Pathology

Ovarian fibrosarcomas are difficult to diagnose clinically and histologically [5]. The final diagnosis of this tumor depends on the postoperative histopathological results because a preoperative diagnosis is relatively difficult. On pathological examination, the specimens are mostly smooth and lobulated, with gray-white sections, a soft texture and a fleshy shape. Focal hemorrhage and necrosis are often seen inside the tumor, as are infiltrative margins that form adhesions with other pelvic organs. Microscopically, the tumor cells were spindle-shaped and exhibited moderate to severe atypia, with large hyperchromatic nuclei of various shapes. The transformation of fibroma into fibrosarcoma could be seen in a few tumors [6].

The malignant potential of ovarian fibrosarcomas is usually assessed based on observed growth patterns, cellular atypia, and mitotic counts. According to previous reports [6, 7], the mitotic count is the most important criterion for the diagnosis of ovarian fibrosarcoma: mitotic counts ≤ 3/10 HPFs indicate benign fibromas, while mitotic counts ≥ 4/10 HPFs indicate fibrosarcomas. However, it has also been reported that patients with high mitotic counts have a good prognosis [8], and thus, mitotic counts should be combined with cellular atypia in order to render the final diagnosis. Lin et al. suggested that ovarian fibrous tumors should be classified based not only on mitotic counts but also on tumor size, growth rate, and the Ki-67 proliferative index [9]. Therefore, for tumors with mitotic counts ≥ 4/10 HPFs but without severe nuclear atypia or other risk factors, mitotically active cellular fibroma should be considered [8, 9]. To avoid excessive treatment, especially in young nulliparous women, complete resection of the ovarian tumor is sufficient, and adjuvant chemotherapy is not necessary [9].

The immunohistochemical profile is not specific to ovarian fibrosarcoma, but it could help in the pathological diagnosis. According to previous reports, Ki-67 can reflect the proliferative activity of tumor cells, and its positive rate seems to be consistent with the degree of malignancy and the prognosis of the tumor. The highest positive rate of Ki-67 was 70% in case 5, and of all the cases, this patient experienced the most rapid tumor progression. Her tumor quickly relapsed 5 weeks after the first surgery and 3 weeks after chemotherapy. Specifically, Ki-67 can be used as an indicator, especially in cases with mitosis, such as 3–4 mitotic areas/HPF, and has recently become an important indicator that can assist in the diagnosis [3, 10]. In addition, expression of Vimentin, α-inhibin, SMA, estrogen receptor (ER) and progesterone receptor (PR) may also be positive [5].

Sometimes, intraoperative frozen pathology sections may indicate spindle-cell malignant tumors, but no cases confirmed by fast-frozen sections have been reported, and consequently, paraffin-embedded sections and immunohistochemistry are often required for a final diagnosis [10]. Since ovarian fibrosarcoma is uncommon and can resemble other malignant spindle-cell tumors, the criteria for differentiation are not clear, especially between mitotically active fibromas and fibrosarcoma. Therefore, ovarian fibrosarcoma is easily misdiagnosed when intraoperative frozen sections are used for pathological examinations, which leads to the administration of inappropriate therapy. In our cases, intraoperative rapid pathological diagnosis in case 2 indicated follicular fibroma. In case 5, intraoperative frozen section analysis showed a spindle cell tumor with a large necrotic area, which was considered a sex cord-stromal tumor, but a malignant tumor was not excluded. Based on our experiences, we suggest that gynecological surgeons be cautious when a diagnosis is made by frozen sections and to wait for the final results of extensive sampling. To account for the possibility of a malignant tumor, gynecologists should evaluate other parameters intraoperatively, such as capsular disruption, necrosis, or adhesion, which are signs of a possible malignancy.

### Treatments

In contrast to epithelial tumors, currently, no standard treatments for the management of ovarian fibrosarcoma have been widely accepted. In case 1, hysterectomy plus bilateral adnexectomy was performed. The patient was administered 2 cycles of systemic chemotherapy consisting of Cisplatin + Vincristine + Bleomycin (PVB) at 3-week intervals. No elevated CA125 level or recurrence was seen 8 years after treatment. For case 2, ovarian cystectomy and sampling of the right ovary were performed during surgery without postoperative chemotherapy. A secondary cytoreductive surgery was performed in case 3, and the tumor was completely removed, after which chemotherapy was given again. In case 4, the left adnexa was removed without chemotherapy after surgery because of her medical complications and older age. Specifically, total hysterectomy and bilateral adnexectomy were performed as the first surgery in case 5. Then, we performed a second surgery 5 weeks after the first surgery and 3 weeks after PEI chemotherapy in which the pelvic mass was partially removed. According to previous studies, early and thorough surgery is preferred, but the surgical methods differed significantly in reports of individual cases; surgical methods ranged from unilateral adnexectomy to complete cytoreductive surgery (hysterectomy + bilateral adnexectomy + omentectomy + appendectomy + pelvic and/or abdominal lymphadenectomy) [11–13]. During surgery, comprehensive exploration and surgical staging are necessary, and cytoreduction should be performed as completely as possible. For young patients with fertility requirements, whether fertility-preserving surgery should be performed is somewhat controversial.

After surgery, adjuvant radiotherapy and chemotherapy should be selected on an individual basis. Due to the high degree of malignancy and the high recurrence rate, the prognosis of ovarian fibrosarcoma is very poor. Few studies have reported which adjuvant chemotherapy improved the survival rate of patients with fibrosarcoma, and thus, it is recommended that even patients with early-stage disease should also be given chemotherapy [3]. However, no unified standard regimen has been established for ovarian fibrosarcoma. Previous case reports included various combined regimens, such as adriamycin + ifosfamide + Methina + azenimide (MAID), adriamycin + ifosfamide (IA), paclitaxel + cisplatin (TP), cisplatin + vincristine + bleomycin (PVB), among others [1, 10–12]. It has also been reported that the combination of paclitaxel and cisplatin is an effective regimen that improves the survival rate after cytoreductive surgery [9, 10]. Therefore, at present, no consensus on surgical and adjuvant therapies has been established. In our cases, two patients refused chemotherapy due to their older age and medical complications. Younger patients in their forties and fifties did not receive appropriate chemotherapy because of personal choice. The last patient (case 5) abandoned all treatments because her tumor relapsed quickly, and we could not offer a better solution.

### Prognosis

Currently, the treatments and prognostic factors of ovarian fibrosarcoma are still debatable. Although several cases of long-term survival have been reported [14, 15], the prognosis of ovarian fibrosarcoma is still generally poor. According to previous reports, the OS of patients is generally less than two years due to resistance to adjuvant chemotherapy and early metastasis via the bloodstream and tumor recurrence [3, 5]. Studies have shown that patients with different FIGO stages, Ki-67 indexes and treatments may have different survival rates [13]. Patients with stage I disease (77.1% vs. 36.2%, *P* = 0.014) and a positive rate of Ki-67 < 10% (100% vs. 27.2%, *P* = 0.042) have a better 2-year survival than other patients. From our experiences, the positive rate of Ki-67 may be consistent with the degree of malignancy and prognosis of the tumor. In our cases, the highest positive rate of Ki-67 was 70% in case 5, and the tumor quickly relapsed 5 weeks after the first surgery and 3 weeks after chemotherapy. We performed a second surgery, which involved partial removal of the pelvic mass. The overall survival was less than 1 year.

In our cases, there were significant differences in the surgical methods, which ranged from ovarian cystectomy to secondary cytoreductive surgery. In addition, no unified chemotherapy existed for the above 5 cases, and of these, two patients even refused chemotherapy due to older age and medical complications. Younger patients in their forties and fifties did not receive the appropriate chemotherapy because of personal choice. The last patient (case 5) abandoned all treatments because her tumor relapsed so quickly, and her tumor was not sensitive to chemotherapy. Unfortunately, we could not offer a better solution. The overall survival rates were > 15, >7, > 6, <1, and < 1 year for each patient. The first patient had the best postoperative outcomes with very rare long-term survival. The reason for this seems to be that the sarcoma was well-differentiated, at an early stage (IC) and was completely removed, although not enough cycles of chemotherapy were administered.

As for the significance of how the treatment method affects the prognosis, it has been reported that patients who received complete cytoreductive surgery combined with adjuvant chemotherapy had better survival outcomes than others, such as patients who received only unilateral adnexectomy or surgery plus radiotherapy (100% vs. 27.9%, *P* = 0.002) [11, 13]. In the multivariate analysis, FIGO staging and treatments were independent prognostic factors, while age, tumor size, mitotic counts and other factors were not statistically significant [3, 13]. However, different chemotherapy regimens were not included in the statistical comparison because of the small number of cases. Since the number of reported cases with ovarian fibrosarcoma is so small and the experiences are so limited, it is difficult to enroll enough patients needed to evaluate the prognostic factors; as a result, standard treatments have not yet been established. Previous reports have all recommended radical surgery with staging and complete cytoreduction, after which patients could have a better prognosis and longer survival with or without chemotherapy or radiotherapy [13].

## Conclusions

Ovarian fibrosarcoma is a rare ovarian malignant tumor with a poor prognosis, and the diagnosis of this tumor should be based on postoperative pathology rather than on intraoperative frozen sections. Patients with an early disease stage and low positive rate of Ki-67 staining have a better prognosis than other patients. At present, evidence-based large-scale case studies are lacking, but nevertheless, we suggest that complete cytoreductive surgery is essential. For adjuvant therapy, regimens used for malignant cord stromal tumors, as stated in the NCCN guidelines, are recommended. Due to the low incidence of ovarian fibrosarcoma, multicenter clinical and molecular studies are required to elucidate its clinical features and to discover new treatments that lead to a better prognosis.

## Data Availability

The dataset that supports the cases presented in this article is included within the article as in Table 1.
